# Surface Modification of Biomedical Ti and Ti Alloys: A Review on Current Advances

**DOI:** 10.3390/ma15051749

**Published:** 2022-02-25

**Authors:** Jingyuan Xu, Jiawen Zhang, Yangfan Shi, Jincheng Tang, Danni Huang, Ming Yan, Matthew S. Dargusch

**Affiliations:** 1 School of Mechanical and Mining Engineering, The University of Queensland, Brisbane 4072, Australia; jingyuan.xu@uq.edu.au; 2 Department of Materials Science and Engineering, Southern University of Science and Technology, Shenzhen 518055, China; 12032310@mail.sustech.edu.cn (J.Z.); 11811137@mail.sustech.edu.cn (Y.S.); 12149007@mail.sustech.edu.cn (J.T.); danni.huang@uq.edu.au (D.H.)

**Keywords:** additive manufacturing, Ti and Ti alloys, biomaterial, surface modification, implant

## Abstract

Ti is widely used as a material for orthopedic implants. As rapid and effective osseointegration is a key factor for the successful application of implants, biologically inert Ti materials start to show inherent limitations, such as poor surface cell adhesion, bioactivity, and bone-growth-inducing capabilities. Surface modification can be an efficient and effective approach to addressing the biocompatibility, mechanical, and functionality issues of the various Ti implant materials. In this study, we have overviewed more than 140 papers to summarize the recent progress in the surface modification of Ti implants by physical and/or chemical modification approaches, aiming at optimizing their wear resistance, biocompatibility, and antimicrobial properties. As an advanced manufacturing technology for Ti and Ti alloys, additive manufacturing was particularly addressed in this review. We also provide an outlook for future research directions in this field as a contribution to the development of advanced Ti implants for biomedical applications.

## 1. Introduction

Among metals and alloys, Ti and Ti alloys exhibit superior biocompatibility, chemical inertness, and mechanical properties. They have been extensively used for medical implants intended for human bones and joints, and as dental implants. The titanium alloys that have been widely used in commercial biomedical devices are commercial pure (CP) Ti and Ti-6Al-4V, of which CP Ti is mainly used in fields requiring better biocompatibility such as dentistry, while Ti-6Al-4V is mainly used in load-bearing applications. Other new developmental α+β [1] and β Ti alloys [2,3] enable avoiding the use of Al, V, and other elements that are harmful to the human body [4,5]. Their elastic moduli are also relatively low, which is beneficial to minimize the “stress shielding” effect caused by excessively high elastic moduli of previous Ti materials.

**Figure 1:**
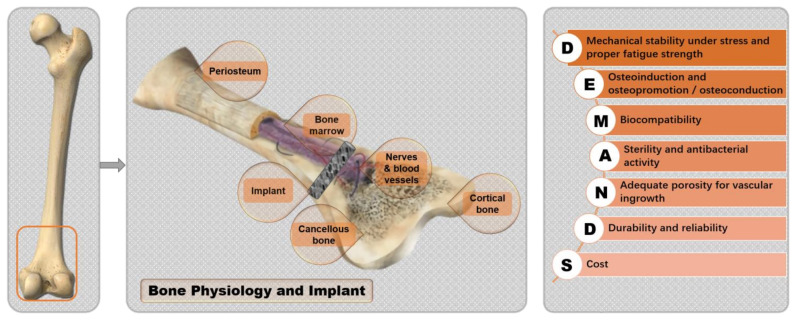
Bone anatomy and implant requirements modified based on Refs. [8,9,16].

Despite the advantages, Ti is a biologically inert material [6]. The biological activity of Ti-based alloys and their ability to induce bone growth are inadequate, and the rate of osseointegration after implantation in the human body is slow [7]. In this regard, Figure 1 shows the physiological structure of human bones [8,9]. The inorganic substance of bone is mainly phosphate. The dense oxide film that forms on the surface of Ti materials is unfavorable for inducing calcium phosphate deposition in the body [10], causing inadequate biological integration between the surrounding bone tissue and the implant, which would normally be essential to achieve early and firm osseointegration [11]. This film and its impact on biological integration may result in poor bonding and osseointegration, leading to implant loosening and shedding. Thus, surface treatment is generally required to improve the biocompatibility of Ti alloy implants.

Figure 1 also lists the ideal properties of bone implant materials. The surface characteristics of Ti and Ti alloys determine their corrosion resistance, wear resistance, and biocompatibility [12]. In recent years, Ti alloy implants prepared using cutting-edge technologies have exhibited complicated structures [13], such as hierarchical porous morphology, on the surface or throughout the implant. To ensure the durability and reliability of the implants in the environment in which they are applied, new surface modification methods and a variety of manufacturing technologies have been developed and implemented. Among the advanced manufacturing technologies, three-dimensional (3D) printing and additive manufacturing (AM) can be used for substrate alloy preparation [14,15], surface modification, or coating manufacture.

**Figure 2:**
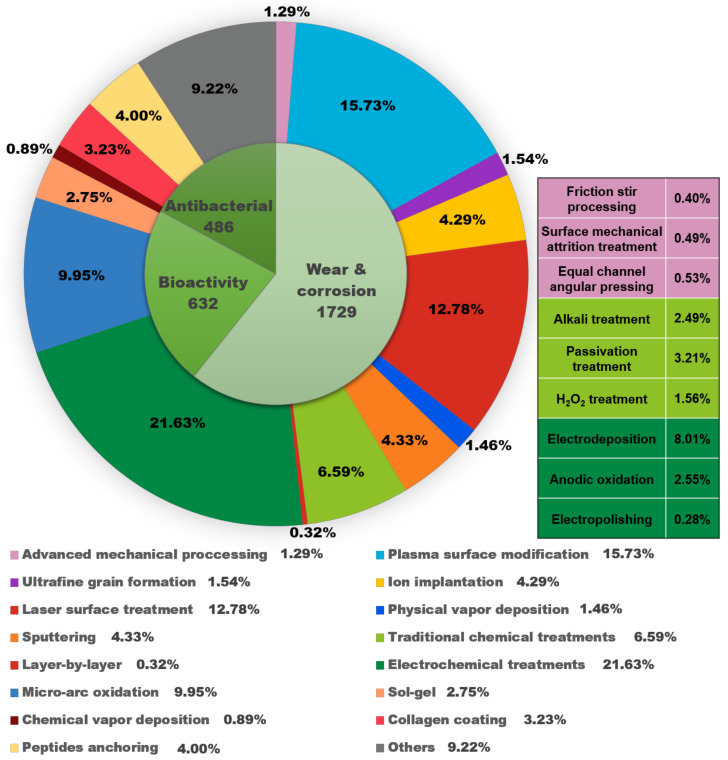
Schematic diagram of the statistical results of research papers on the topic of surface modification of Ti alloys in the Web of Science Core Collection in the past three years. Inner circle: number of papers in different topics; outer circle: percentage of papers on different surface modification technologies. Attached table (corresponding to colors): number of papers for different treatments in advanced mechanical processing, traditional chemical treatments, and electrochemical treatments.

Figure 2 further summarizes the research papers on surface modification of Ti alloys published in the Web of Science Core Collection over the past three years. Among the >2000 papers, over 1700 are concerned with wear and corrosion properties, and more than 600 and nearly 500 papers are dedicated to improving bioactivity and antibacterial properties, respectively, suggesting that these are the three most important aspects for enhancing the biomedical performances of Ti and Ti alloys. 

This article reviews the recent progress of several surface modification approaches for developing advanced biomedical Ti and Ti alloys, including the latest 3D printing technology. An overview of biomedical Ti alloys is presented in the first place, followed by surface modification achievements for improving wear, bioactivity, and antibacterial properties, as shown in Figure 2. Finally, suggestions for related future research are proposed, with the aim of providing a reference for further research directions and innovative ideas.

## 2. Overview of the Development of Medical Ti Alloys

### 2.1. Brief Introduction of Biomedical Ti and Ti Alloys

During the 1940s, Ti started to be applied as a medical implant material, owing to its low density, excellent mechanical properties, and non-toxicity. In the 1960s, after Brånemark [17] used pure Ti as a dental implant material, this material began to gradually replace stainless steels and CoCr alloys as a biological dental implant material. Since then, research and development of medical Ti alloys has advanced. Ti and its alloys exhibit good biocompatibility, primarily because their surface reacts with oxygen to form a dense TiO_2_ oxide film [18].

Moreover, the dielectric constant of TiO_2_ is similar to that of water [19]. This is because the electrostatic force generated by the oxide film on the Ti surface is relatively weak. In a biological environment, it is difficult for the surface of a Ti implant to adsorb protein molecules [20]. In addition, the isoelectric point (IPE) of TiO_2_ is ~6.2 [21], which is slightly lower than the physiological pH (=7.4). This implies that the negative charge on the Ti surface is relatively weak in the physiological environment, an important factor affecting the osseointegration between the implant and the surrounding living bone [22,23].

With time, the first-generation of α-type pure Ti [24] and Ti-15Zr [25], and α+β-type Ti-6Al-4V alloys [26] gradually developed into a second-generation α+β-type Ti alloys with improved corrosion resistance and strength. Representative α+β-type alloys are Ti-5Al-2.5Fe [2,27] and Ti-6Al-7Nb [2,28]. However, the elastic moduli (60–120 GPa [29,30]) of second-generation Ti alloys are considerably higher than that of the human cortical bone (mostly 10–20 GPa) [31]. When the elastic moduli of the implant and bone tissue are not consistent, the majority of stress is absorbed by the alloy, owing to its higher elastic modulus, and resulting in bone atrophy and stress shielding [32,33]. Consequently, loosening of the implant and bone tissue injury [34] can occur, increasing the possibility of secondary fractures after recovery [35]. Research and development of Ti alloys for medical use is now largely focused on the so-called third generation β models, which have elastic moduli close to that of human bones. This type of Ti alloy does not incorporate harmful or allergenic elements, such as Ni. For example, Plaine et al. [36] developed Ti-35Nb-7Zr-5Ta, which exhibited favorable biological activity and a low elastic modulus (50 GPa), based on Niinomi’s Ti-29Nb-13Ta-4.6Zr [2]. Furthermore, a new Ti-24Nb-4Zr-7.9Sn alloy (Ti2448) has been developed by the Chinese Academy of Sciences. Its elastic modulus is merely 45 GPa, with 850 MPa tensile strength, and superior corrosion resistance [37,38,39].

### 2.2. Significance of Surface Modification

Ti and its alloys are biologically inert materials, and their structures and properties are distinct from those of bone tissue materials. They cannot be directly chemically bonded to bone tissue in the human body and the formation of a fibrous tissue layer at the bonding interface is common, thus loosening the implant [40]. In addition, wear debris causes inflammation, and the commercially mature Ti-6Al-4V implants may release toxic Al and V ions into body fluids [41,42,43]. In practical applications, the degradation of Ti and Ti alloys invariably starts from the surface, necessitating the synthesis of a protective or biocompatible layer on the surface of Ti and Ti alloys [44]. At present, surface modification of medical Ti alloys generally involves depositing a compatible coating on the surfaces, or changing the structure and composition of the surfaces to directly improve corrosion and wear resistance, biocompatibility [45], antimicrobial properties, and other properties [46]. The improvement of existing bioactive coating preparation methods and further exploration of new bioactive coating materials are vital to achieving high-quality bioactive surface modification for biomedical Ti and Ti alloys [47].

### 2.3. Brief Introduction of Current Advances of Surface Modification Technologies

As shown in Figure 2, recently researchers have studied various advanced methods to modify the surface of Ti and Ti alloys, such as ultra-grain refinement, physical vapor deposition (PVD), micro-arc oxidation (MAO), electrochemical treatments, etc. [48,49,50,51,52,53]. Table 1 lists several commonly used Ti and Ti alloys, along with research works on their surface modification.

Regarding biomedical applications of Ti alloys, in addition to the preparation of implants with various mechanical properties and distinct lattice structures [54], layered processing is also often used for surface treatment [55]. It is extensively implemented in medical biology owing to its unique merits such as high production efficiency, short processing time, low processing cost, and ease of customization [56]. As a latest layered processing technology, AM is used to modify the surfaces of biomedical Ti alloys as well. Among the AM techniques, powder bed fusion (PBF) is mostly based on laser, for instance, the selective laser melting (SLM) [57,58], or electron beam. Biomedical Ti implants fabricated using PBF can have a sophisticated geometry and complex internal structure, which may require more advanced surface modification techniques. In contrast, another laser-based AM technique, the laser engineered net shaping (LENS™), uses powder deposition system (often via coaxial nozzle) rather than powder bed [59,60]. It typically applies a higher laser power and a larger spot size than SLM, which makes its deposition rate and working temperature both higher but a lower resolution and geometry accuracy [61]. Comparatively speaking, LENS™ is more often used for coating/surface modification purposes.

## 3. Surface Modification for Biomedical Ti and Ti Alloys

### 3.1. Overview on Surface Modification Techniques for Improving Wear Resistance

**Figure 3:**
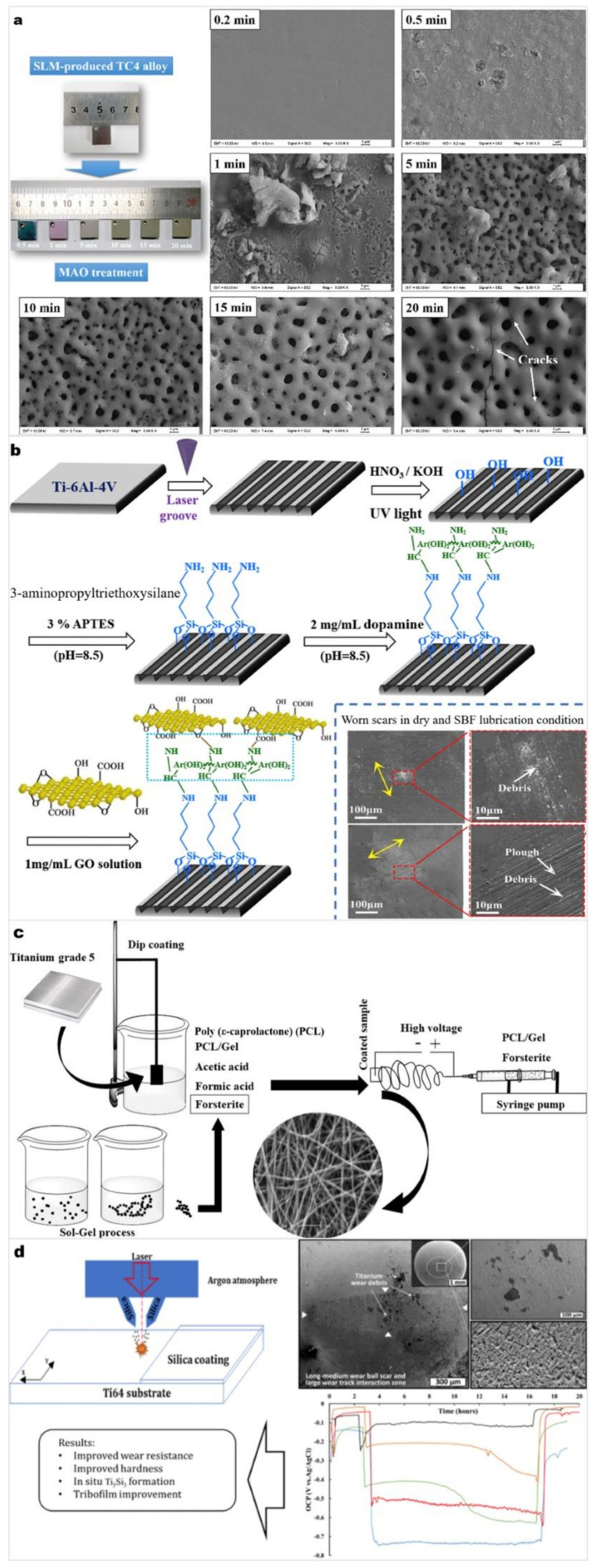
(**a**) Scanning electron microscopy images of the Ca/P-containing titania coatings produced by MAO on SLM-produced Ti64. (**b**) Graphene oxide (GO) coatings grafted on the laser microgrooved Ti64 surfaces with the combination of 3-aminopropyltriethoxysilane (APTES) and dopamine adhesion layers. (**c**) Poly (ε-caprolactone) (PCL)- Gelatin (Gel)-forsterite nano composite coating on CP Ti. (**d**) SiO_2_ coating deposited on Ti64 by SLM technology. Panel (**a**) reprinted with permission from [69] (Copyright 2019 Elsevier). Panel (**b**) reprinted with permission from [70] (Copyright 2020 Elsevier). Panel (**c**) reprinted with permission from [71] (Copyright 2020 Elsevier). Panel (**d**) reprinted with permission from [72] (Copyright 2021 Springer Nature).

Low surface hardness, high friction coefficient, and poor wear resistance are typical shortcomings of Ti and Ti alloy implants. At present, a variety of methods for improving the wear performance of Ti alloy surfaces have been developed, including changing the surface roughness or depositing a ceramic coating with excellent wear resistance on the Ti surface to improve its wear and corrosion properties [62]. The most commonly used treatments for the surfaces of Ti alloys are polishing [63], grinding [64], sandblasting [65], and laser etching [66]. These methods mechanically modify the surface to form surfaces with a certain roughness to improve the bonding strength between the substrate and coating. Commonly used wear-resistant coatings are diamond-like carbon (DLC) [67] and Ti nitride (TiN) [68]. Figure 3 lists several research advances of coating technologies to improve the wear and corrosion performance of Ti and Ti alloys [69,70,71,72]. The adopted methods include micro-arc oxidation (MAO), laser grooving, and SLM. In this section, various technologies and their practical applications are described. One of the main opportunities for biomedical Ti and Ti alloys is to improve friction and wear performance. This means that mechanical, physical, and chemical methods are used more frequently. Biochemical surface modification techniques are detailed in the latter two sections, which includes traditional methods such as sandblasting, etching, thermal spraying, plasma treatment, alkaline heating, and anodic oxidation. It also provides examples of some advanced methods such as sputtering, micro-arc oxidation, and PBD. Most of these methods tend to improve the composition along with the type of phases, and detailed microstructure of the alloy surface.

Typical examples are summarized as follows: (a) After plasma treatment, TiN-coated commercially-pure (CP) Ti knee and hip implants showed significant changes in surface texture and wettability, with improved biological compatibility and wear resistance [73]; (b) The main advantage of anodized films on Ti alloys is their denseness and rigidity, improved adhesion and wear resistance [12,74]. Wu et al. [75] generated a TiN nanotube array coating via ammonia-mediated reduction and nitridation of TiO_2_ nanotubes (TNTs) prepared via anodic oxidation. This ensured that the porous structure meets the requirements of biocompatibility but also improved the wear resistance; (c) as the principal inorganic component of human bone, hydroxyapatite (HA) displays good biocompatibility and can facilitate bone conduction and induction [76]. Sandblasting removes contaminants or plastic deformation layers and obtains a topographically specific surface for further modification. Cao et al. [77] annealed an HA coating via thermal spraying onto the surface of Ti-6Al-4V, and the bonding strength of the coating increased significantly because the heat treatment led to a reduction in residual stress; (d) Thermal spraying requires high-speed spraying of heated HA (and other raw materials) to the substrate to form a coating. Yan et al. [78] used SLM to fabricate Ti64 triply periodic minimal surface lattices, followed by heat treatment, sandblasting, and HCl and NaOH etching, and finally soaking in simulated body fluid (SBF) for 14 days to achieve a dense, uniform surface. The bone-like biomimetic HA layer (derived from the sodium titanate hydrogel layer produced through alkali treatment) had a thickness of approximately 5.6–7.5 μm. 

In the following section, we will further summarize research advances in terms of physical, chemical, and AM approaches for improving wear resistance. 

#### 3.1.1. Physical Technologies for Improving Wear Resistance

**Figure 4:**
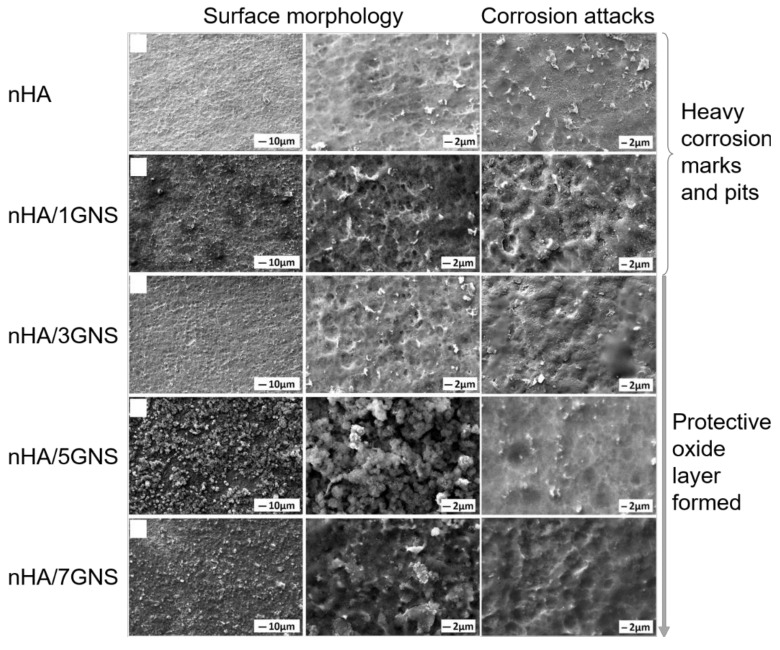
Surface morphology of nHA/GNS coating before and after SBF corrosion tests. Figure reprinted with permission from [80] (Copyright 2020 Elsevier).

Examples of physical surface modification methods include physical vapor deposition (PVD) and sputtering. The PVD coating obtained by evaporation or sputtering of the source material in a high vacuum has high density and strong adhesion to the substrate. Kao et al. [79] nitrided selectively-laser-melted Ti64 at a high temperature of 900 °C, and used closed-field unbalanced magnetron sputtering to deposit Ti-doped DLC (Ti-C:H) thin films. Potentiodynamic polarization and reciprocating sliding wear tests indicated considerably better corrosion resistance (1.93-fold lower corrosion current was observed compared to that of unmodified samples), lower wear depths (35–114 times lower, depending on the sliding ball used), and lower friction coefficients. Yigit et al. [80] prepared a nano-HA (nHA) coating doped with graphene nanosheets (GNSs), and obtained a nanoscale porous morphology, as shown in Figure 4. As the mass fraction of GNS in the hybrid structure increased, the number of nHA nucleation sites in the coating increased significantly, thereby enlarging the surface area and enhancing the corrosion resistance of the surface. Singh et al. [81] examined the addition of TiO_2_, Y_2_O_3_, Al_2_O_3_, polyethylene, chitosan, yttrium stabilized zirconia, Ni_3_Al, and carbon nanotubes (CNT) to HA coatings as reinforcement materials. The hardness and toughness of these composite coatings was superior to that of pure HA coatings, and subsequent heat treatment improved the structural properties and bonding strength of the composite coating.

#### 3.1.2. Chemical Technologies for Improving Wear Resistance

**Figure 5:**
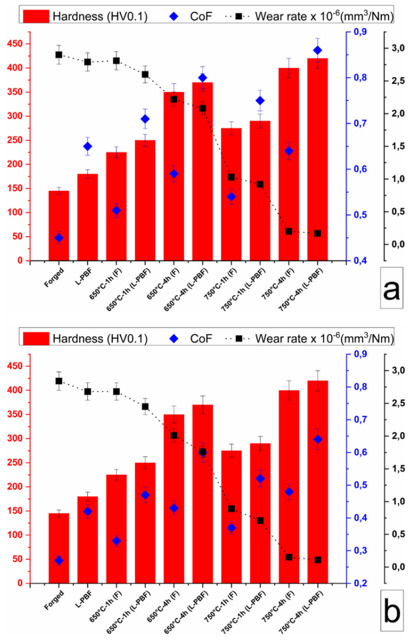

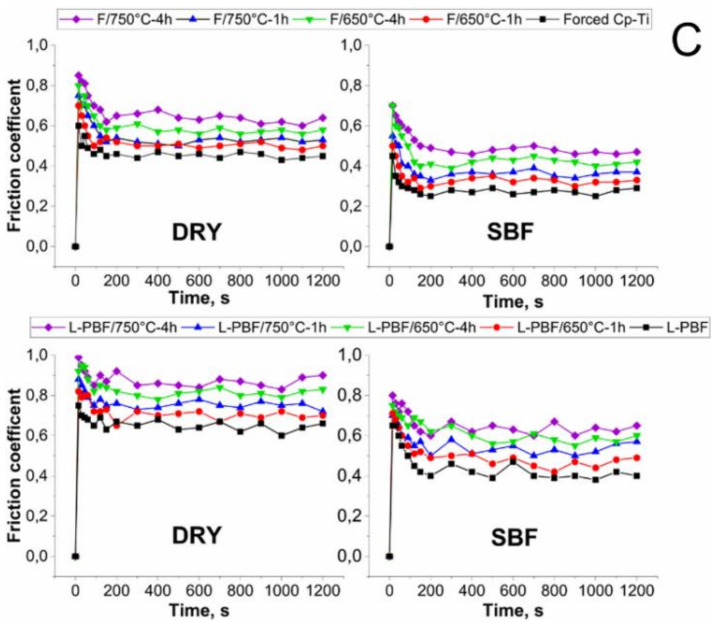
Hardness-friction coefficient-wear rate graphs of forged and SLM samples: (**a**) dry and (**b**) SBF conditions. (**c**) Friction coefficients of samples under dry and SBF conditions (L-PBF represents SLM). Figure reprinted with permission from [88] (Copyright 2019 Elsevier).

MAO is an advanced chemical process derived from anodic oxidation [82]. In this surface treatment technology, a Ti alloy is used as an anode in an electrolyte and a high voltage is imposed between it and an inert cathode to produce a micro-arc discharge on the surface of the material, generating an oxide film in situ on the surface of the Ti alloy [83]. In contrast to other methods, a TiO_2_ layer is generated both on the surface of the material and under the surface to generate a diffusion region, thus improving the surface bearing capacity [84,85]. Hu et al. [86] employed MAO to modify Ti64; the hardness of the alloy surface film (500–800 HV) was approximately 2–3-fold higher than that of the internal matrix (approximately 350 HV), and the wear resistance was considerably improved. Qi et al. [87] micro-arc oxidized Ti64 in NaAlO_2_, and the resulting ceramic film hardness was as high as 1140 HV, which significantly improved the poor wear properties of the alloy, enabling its application in friction portions of medical instruments. Micro-arc-oxidatively forged and selectively laser melted CP Ti samples were prepared by Kovacı [88], and their structural, mechanical, and tribological properties were characterized and measured under both dry and SBF conditions. The diffusion zone depth of selectively laser-melted CP Ti was greater than that of the forged CP Ti. Figure 5 shows the hardness and friction properties of various types of samples, where it is observed that selectively-laser-melted samples with MAO at 750 °C/4 h exhibited the best performance. Hu et al. [89] attempted to incorporate ultrasonication into the MAO technology to reduce the pore size (3–8 μm) on the surface of the coating, generate a more uniform and compact structure, and enhance the binding force with the substrate, corrosion resistance, and mechanical and biological properties of the coating. The corrosion current of ultrasonic MAO-treated CP Ti reduced by 72% when compared with untreated samples. The results of the above studies are qualitatively the same: high thickness and hardness, and ceramic-like structural strength, even if different electrolyte compositions and energization parameters are adopted by different researchers.

Other chemical approaches include alkali heat treatment and electrospinning. Chen et al. [90] used induction heating combined with alkali treatment to prepare a stable layered micro-nanoporous network structure on the surface of Ti, resulting in a more uniform coating surface with a higher number of pores, and improved roughness, wettability, and bonding strength with the matrix, as well as significantly improved in vitro and in vivo biocompatibility properties. Manole et al. [91,92] deposited n-type semiconducting TiO_2_ nanowires onto a Ti-50Zr surface by electrostatic spinning and improved the hydrophilic and corrosion behavior of the alloy surface whilst avoiding significant inflammatory processes. The protection efficiency of the coatings remained greater than 51%.

#### 3.1.3. 3D Printing Technologies for Improving Wear Resistance

The AM technology, LENS™, can directly create a coating on the surfaces of alloys. This line-of-sight process is applied to both flat and curved surfaces to improve the wear and corrosion resistance of implant surfaces. In this section on surface modification using LENS™, the aspects of LENS as an additive manufacturing process are not reviewed, but its capabilities for coating/surface additions are discussed.

**Figure 6:**
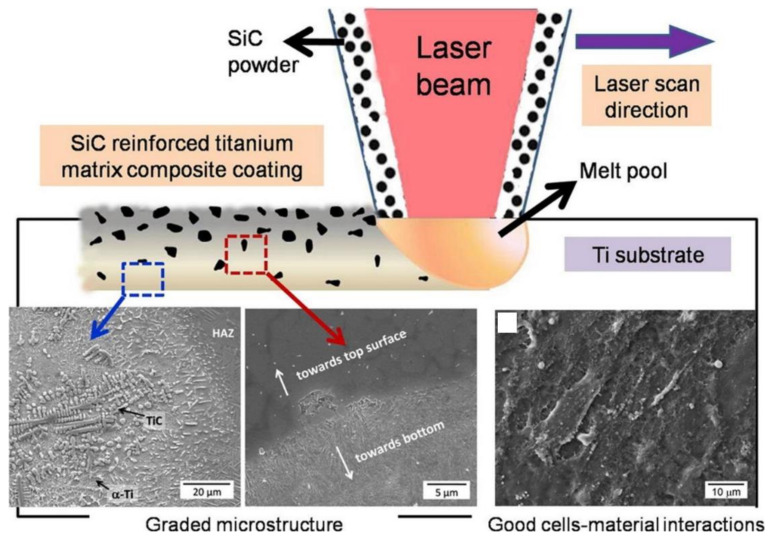
Preparation process and properties of SiC reinforced TMC. Figure reprinted with permission from [95] (Copyright 2016 Elsevier).

Ke et al. [93] employed LENS™ to deposit a Ti-6Al-4V interface layer mixed with 3% HA on a Ti-6Al-4V substrate, followed by plasma-sprayed HA coating. This gradient HA coating improved the bond strength resulting from plasma spraying by introducing a thermal barrier, thereby increasing the bond strength between the substrate and the coating. Heer et al. [94] used LENS™ to directly deposit silica coating on the surface of CP Ti, and the in situ formation of a friction film was observed in the wear test, which indicated that the material had self-healing properties. In addition to an excellent hardness of 1500 HV, this material also exhibited favorable electrochemical properties without biological toxicity. Das et al. [95] used a high-power laser to create a Ti melt pool, and then injected SiC powder to develop a SiC-reinforced Ti matrix composite (TMC). This composite coating was layered on CP Ti implants via LENS™, whereby the reinforced ceramic phase improved the wear (~100-fold) and corrosion resistance of Ti. Such metal-ceramic composite coatings, manufactured as shown in Figure 6 [95], improve strength, hardness, and subsequent wear resistance, and can be applied to the surfaces of load-bearing implants, such as joint replacements, to extend their lifespan in the body by reducing the release of metal ions. Another similar example is the application of LENS™ by Bandyopadhyay et al. [96] to coat CaPTi composites on the Ti surface. The wear resistance of the sample was enhanced because CaP was preferentially worn on the implant surface and a friction film was formed. Stenberg et al. [97] used LENS™ to deposit an extremely dense Ti64 coating consisting of CNTs and CaP ceramics on CP Ti, increasing the hardness of the coating and reducing the wear rate. The increase in the hardness was due to the CNTs facilitating in-situ carbide formation. This coated layer effectively reduced the release of Ti ions and ultimately improved biocompatibility. When researchers use LENS for coating preparation, due to its high processing efficiency and ability to handle complex coating materials, the surface hardness of modified Ti and Ti alloys can be increased by 3–5 times, and the wear resistance can be improved by 30–100 times.

### 3.2. Overview on Surface Modification Techniques to Improve Biological Activity

**Figure 7:**
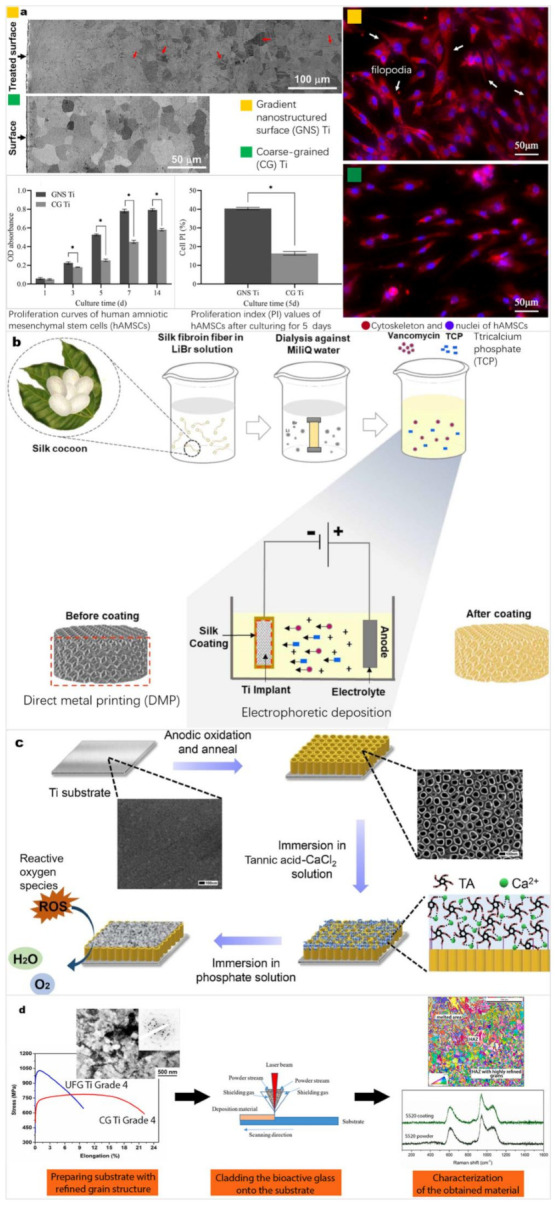
(**a**) Gradient nanostructured surface CP Ti were prepared by surface mechanical attrition treatment, and coarse-grained CP Ti by the followed recrystallization annealing. (**b**) Tricalcium phosphate (TCP)/vancomycin-loaded silk coating on a 3D printed porous Ti substrate. (**c**) HA/tannic acid composite coating based on Ti substrates modified by TNT arrays. (**d**) S520 bioactive glass coating on ultrafine-grained (UFG) CP Ti by laser cladding. Panel (**a**) reprinted with permission from [101] (Copyright 2020 John Wiley and Sons). Panel (**b**) reprinted with permission from [102] (Copyright 2020 IOP Publishing). Panel (**c**) reprinted with permission from [103] (Copyright 2020 Elsevier). Panel (**d**) reprinted with permission from [104] (Copyright 2021 Elsevier).

In the early stages of implantation, the osseointegration capacity of Ti and TiO_2_ surfaces is insufficient, which leads to poor differentiation of osteoblasts and formation of fibrous tissue around the implants [48], further resulting in loosening of the implants and inflammation due to friction and other conditions. To improve the biocompatibility of materials and their osseointegration capacity, it is necessary to construct an appropriate coating on the alloy surface to improve the surface wettability [98] and to create a specific surface morphology that is conducive to cell adhesion, proliferation, and differentiation, and enables protein adsorption. Therefore, adjustment of the surface structure and composition of Ti alloys is necessary, while maintaining their corrosion resistance and mechanical properties. Electrochemical and biochemical techniques can be used to introduce biologically active substances other than titanium alloy and titania as coatings after the use of acid etching, shot peening, and other methods to modify the structure of the surface on the titanium alloy to include nano-size features. At present, common surface modifications of Ti alloys of bone implantation include HA [99], chitosan [100], and TNT array coatings. Among them, TNT arrays can often be combined with other coatings to improve functionality because they present an in situ self-growing porous structure. Figure 7 shows some surface modification studies to improve the osteoconductivity and cell viability of Ti [101,102,103,104]. Electrochemical techniques such as electrodeposition, electropolymerization, electrophoretic deposition, and electrothermal polarization can prepare a variety of highly bioactive coatings. Anodic oxidation and MAO can prepare TiO_2_ nanotubes and coatings with microporous oxide films, respectively. LENS™ can also melt Ti surfaces to form highly adsorbable coatings of different compositions with its unique precise rapid-cooling deposition.

Hydrogen peroxide can react with Ti and Ti alloys to produce Ti–peroxyl gels, which facilitate apatite deposition on Ti surfaces and are beneficial for orthopedic implants. The important result in terms of mechanical techniques is the creation of a nanocrystalline work-hardened surface layer. Due to the better bioaffinity of nanoscale Ti grains with bone cells, this modification that creates nano/submicron surface grains promotes osteogenesis and reduces infection. This can occur either alone or in synergy with coatings incorporating different functions using nano-sized bioactive substances.

Huang et al. [105] confirmed that the surface roughness of a Ti-based implant determines the reactivity between the implant and bone at their interface and directly affects the bone cell activity. Therefore, roughening Ti alloy implant surfaces is crucial for osseointegration. Torres et al. [106] used traditional methods to form nanostructures on the surface of selectively-laser-melted Ti64. After acid etching, the sample was chemically treated in H_2_O_2_, followed by heat treatment. The nanostructured surface on the metal implant enhanced osteogenic activity. Employing shot peening, Chen et al. [107] prepared a deformable layer on the surface of Ti64 with a surface gradient nanocrystalline structure and adjustable thickness and grain size. The nanocrystalline structure of the Ti alloy surface produces abundant grain boundaries, improves hydrophilicity, and induces osseointegration more readily. To obtain UFG CP Ti, Bulutsuz et al. [108] sand-blasted the surface of CP Ti processed by equal angular channel pressing, which greatly increased the Hardness and maximum tensile strength (~70%). Under the premise of similar surface roughness, it was found that those surfaces are more suitable for the proliferation of human gingival fibroblasts. 

In the case of HA coatings, a highly dense HA coating is not conducive to cell proliferation and differentiation, and its ability to induce bone formation is limited; thus, it is mainly used as a bone formation scaffold. Compared with micron-sized HA, nano-sized HA has an ultra-fine structure and higher surface activity [109]. Nano-HA is similar to the minerals found in the bone texture of human hard tissues, having a similar chemical and crystal structure [110]. The complexation of nano-HA and CNTs can improve cell proliferation and differentiation [111]. 

Lin et al. [112] formed CaTiO_3_ on the surface of TNTs through vacuum calcification and hydrothermal treatment, providing nucleation points for HA. This method induced chemical bonding between the nano-HA coating and the TiO_2_ matrix, achieving an adhesion strength of approximately 29 N with ~100-μm wide scratch, which increased the osteoconductivity. Fathyunes et al. [113] used ultrasound-assisted pulse electrodeposition to prepare a graphene oxide (GO)-HA coating on TNTs. GO improved the mechanical properties of the HA coatings and was biocompatible. This composite coating increased cell viability and resulted in a faster rate of apatite precipitation. Rafieerad et al. [114] first prepared graphene nanotube films on a Ti-6Al-7Nb substrate via MAO, and then deposited nano-silver particles employing PVD magnetron sputtering. The results of the study showed that a mixture of loaded AgNPs/GO significantly promoted cell adhesion. This method can also be extended to prepare highly complex nanostructured materials with controllable shapes and biological functions for use in various orthopedic operations.

Stoian et al. [115] adopted the method of dripping gentamicin (GS)-containing phosphate-buffered solution (PBS) onto a Ti-50Zr surface to adsorb the drug with nanotubes or nanopores pre-prepared on the surface. The chitosan coating was then covered using dip-coating. Owing to the difference in nanostructure, a mixed layer of GS and chitosan was finally obtained on the surface of the nanopore samples, which resulted in a greater adsorption of the drug in the tube, and the sustained release time was twice that of the nanopore. This indicated that nanostructures under the chitosan layer played an important role in drug release.

In the following section, we further summarize research advances in terms of electrochemical, biochemical, and AM approaches for improving wear resistance.

#### 3.2.1. Electrochemical and Biochemical Methods for Improving Biological Activity

Various electrochemical methods have been used to increase the biological activity of Ti alloys. Classical electrochemical methods, including electropolishing, anodic oxidation, electrodeposition, and electroplating, are heavily researched because of their most cost-effective characteristics. In electrochemical surface modification techniques, Ti alloys are generally used as electrodes, and the results of these modifications are quantified by various factors, including the electrolyte, voltage, and temperature [116]. The high modulus and high-density anodized film improved the corrosion resistance and reduced the ion release.

In improving the biocompatibility of Ti and Ti alloys, Zhang et al. [117] prepared a SiC nanoparticle-reinforced Na and F co-doped HA coating using an electrochemical deposition method. The replacement of Ca^2+^ in HA with Na^+^ promotes cell attachment and bone metabolism, whereas that of OH^−^ ions by F^−^ increases structural stability, stimulates extracellular matrix formation, and enhances osteosynthetic bonding. Therefore, HA coatings co-doped with Na and F (NFH) exhibited excellent biological properties. Qiao et al. [118] used the same method to prepare a Si-Sr-Ag-co-doped HA/TNT coating. Doping with Sr and Si ions enhanced the expression levels of genes related to osteogenesis and successfully neutralized the potential cytotoxicity of Ag ions. The biological properties of co-doped coatings are superior to those of HA and Ag-HA coatings, and their antimicrobial efficacy is similar to that of Ag-HA coatings. Specific high contents of Si and Sr ions can promote the proliferation and regeneration of cells and bone tissues and improve cell adhesion. HA coatings containing Si present a higher absorption rate and contain a large number of active groups for osteoblast attachment. Mumith et al. [119] compared the degree of bone ingrowth and osseointegration of electrochemically coated SiHA and SrHA with that of laser-sintered completely porous Ti64 implants with varying pore sizes. Compared with the structure and preparation method of the bioactive coating and the modulus of the implant, the pore size exerted less influence on osseointegration. Sun et al. [120] studied nanofiber polypyrrole template-free electropolymerization on selectively-laser-melted CP Ti 2D sheets and 3D lattice structures. The obtained coating surface of the lattice had an uneven morphological structure and exhibited poorer reversibility and long-term stability compared to the sheets. An ethanol-based colloidal solution of nanopowder HA was dispersed into a chitosan colloid by Jugowiec et al. [121] and then coated on the surface of Ti-13Nb-13Zr alloy using electrophoretic deposition (EPD). This composite coating resulted in a thicker deposition of chitosan (1.5 μm), displayed robust bonding with the Ti alloy, and improved its corrosion resistance and biocompatibility.

**Figure 8:**
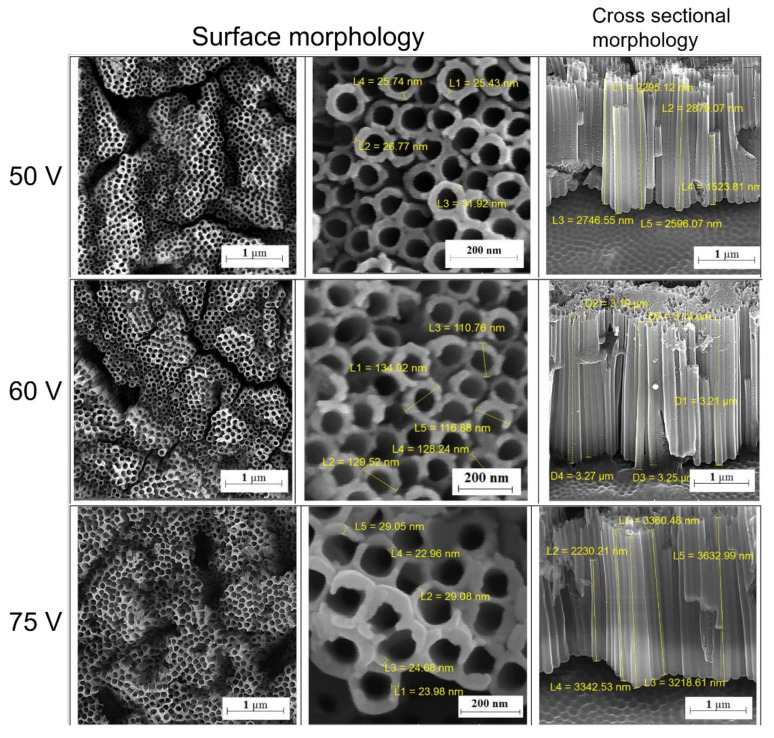
Surface and cross-sectional morphologies of Ti-6Al-4V surface after anodizing process under applied voltages of 50, 60, and 75 V. Figure reprinted with permission from [123] (Copyright 2017 Elsevier).

Anodization and MAO have been applied for the preparation of TNTs and coating of microporous oxide films, respectively. Yu et al. [122] employed electrochemical anodization to prepare TNTs with various morphologies on Ti foils and found that TNTs with a relatively small diameter (30 nm) were more conducive to the adhesion and proliferation of osteoblasts; TNTs with a relatively large diameter (110 nm) exhibited better osteogenic differentiation abilities and osteogenic potential under simulated oxidative stress. Mansoorianfar et al. [123] successfully prepared TNT arrays with good uniformity on Ti-6Al-4V alloy using secondary anodic oxidation at a voltage of 50–75 V. The morphology is shown in Figure 8, wherein it is evident that the average length and diameter of the nanotubes increased with increasing voltage. According to the results of the culture experiment using human osteosarcoma MG63 cells, the samples prepared under a voltage of 60 V showed optimal cell viability. Bandyopadhyay et al. [124] used the electrothermal polarization method to store charges on the TNTs prepared by anodizing the surface of CP Ti. 5 weeks of in vivo experiments showed that the mineralized bone formation around the implants with polarized TNTs surface increased by about 40% compared with TNTs, proving that TNT-P can promote the formation of osteoid and its maturation of mineralized bone. When Shbeh et al. [125] used MAO to treat porous Ti64 portions, the highly porous samples formed a thicker surface coating due to the increase in capacitance. The thicknesses of the coating reached more than three-fold of the coating thickness of relatively dense samples. The porous structure of the sample and the subcutaneous coating network effectively alleviated the stress shielding effect and improved the chemical integration. Wang et al. [126] used MAO to prepare an oxide film on the surface of porous Ti generated via metal injection molding. The microporous structure in the film enabled a high degree of engagement with osteoblasts, and the P and Ca in the ceramic membrane promoted cell adhesion, diffusion, and growth. This can be attributed to their favorable chemical binding with bone matrix proteins, which stimulates biochemical binding between bone tissue and the surface of the implant.

For biomedical applications, Ag and Sr ions can be bound to the electrodeposition film in an electrolytic solution containing dissolved Ag and Sr compounds. Ca and P ions can be bound to the anodic oxide film in an electrolytic solution containing dissolved Ca and P compounds. The non-uniform porous oxide film is obtained by setting the MAO at a voltage higher than the breakdown limit, with more gas escape and frequent sparks occurring in the process [12]. The microporous film and porous substrate prepared by MAO provide an excellent growth environment for osteoblasts. Different osteogenic substances can be introduced by modulating the electrolyte to further improve the bioactivity.

**Figure 9:**
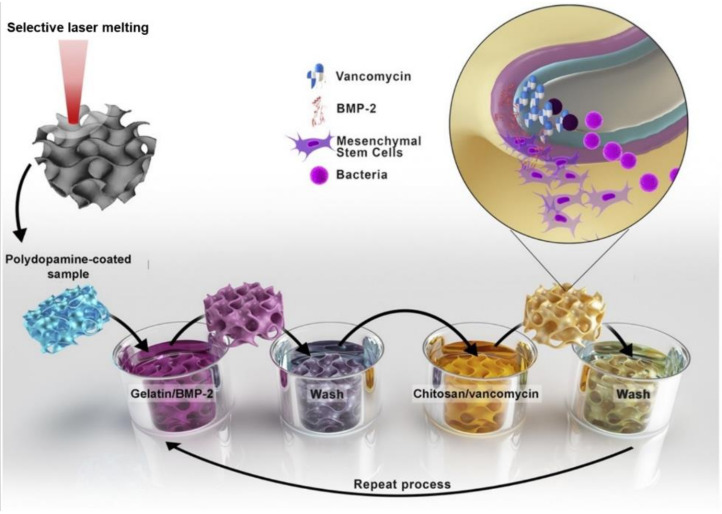
Schematic illustration of the LbL coating process. Figure reprinted under CC-BY license from [129] (2020).

The most extensively utilized method for surface biochemical modification of Ti-based implants is the layer-by-layer (LbL) method, based on the principle of supramolecular electrostatic assembly, in which a self-assembled multilayer biologically active film is formed via alternating adsorption of oppositely charged biologically active macromolecules on the surface of the substrate [127,128]. Yavari’s team [129] successfully employed the LbL method to process drug delivery between micron-scale drug carriers on the surface of a selectively-laser-melted CP Ti scaffold. The growth factor and an antibiotic were introduced into gelatin and chitosan, respectively, followed by immersion of Ti scaffolds into the resultant solutions layer by layer (Figure 9). Thus, both types of active agents were released. In terms of osteogenic activity, samples containing bone morphogenetic protein (BMP)-2, a growth factor, showed exponentially increased mineralization. At the same time, bacterial numbers decreased by eight orders of magnitude. Both BMP and the Arg-Gly-Asp (RGD) peptide are growth factors that can be directly fixed onto the surface of Ti alloy implants. It has been established that BMPs can differentiate into osteoblasts by activating mesenchymal stem cells and promoting collagen synthesis and bone matrix production [130]. Therefore, fixing the bone morphogenetic protein on the surface of Ti alloys can induce the formation of bone tissue on the surface of the implant and accelerate the integration of implant and bone [131]. Heller et al. [132] immobilized the RGD peptide sequence on the surface of a Ti implant to effectively promote the adhesion and differentiation of osteoblasts and accelerate the integration of implant and bone interfaces.

#### 3.2.2. 3D Printing Technologies for Improving Biological Activity

LENS™ can also be utilized to create coatings that promote bone formation. To improve the interaction between bone cells and implant materials, Roy et al. [133] applied LENS™ to melt the upper surface of the Ti substrate and added TCP ceramic powder to form a TCP-Ti composite layer. The coating initiated coating cell differentiation, extracellular matrix formation, and biomineralization. Ke et al. [93] used LENS™ to deposit a 3% HA-Ti64 interface layer on Ti64, and sprayed 1 wt.% MgO-2 wt.% Ag_2_O-HA using plasma to improve the biological and antibacterial properties of the coated implant. The high energy of LENS™ enhanced the crystallinity of the HA layer; thus, the amount of Ag^+^ ions released at the gradient HA LENS™ layer was reduced by 70%, and the osteoconductivity was improved.

Apart from compounds, Ta as metal has extremely high density and high melting point, but it has better biocompatibility than Ti. In order to introduce Ta, Mitra et al. [134] tried to use LENS™ to handle the huge difference in the melting temperature of Ti and Ta. They directly deposited the mixed Ti and Ta powder on the surface of Ti-6Al-4V to form an in situ alloyed low-modulus porous coating. Nanotubes were formed by anodic oxidation. In vivo experiments showed that porous Ti-25 wt% Ta-nanotubes coating has exceptional bone-promoting and biological activity comparable to or even surpassing similar coatings of 100% Ta.

### 3.3. Overview on Surface Modification Techniques to Improve Antibacterial Performance

**Figure 10:**
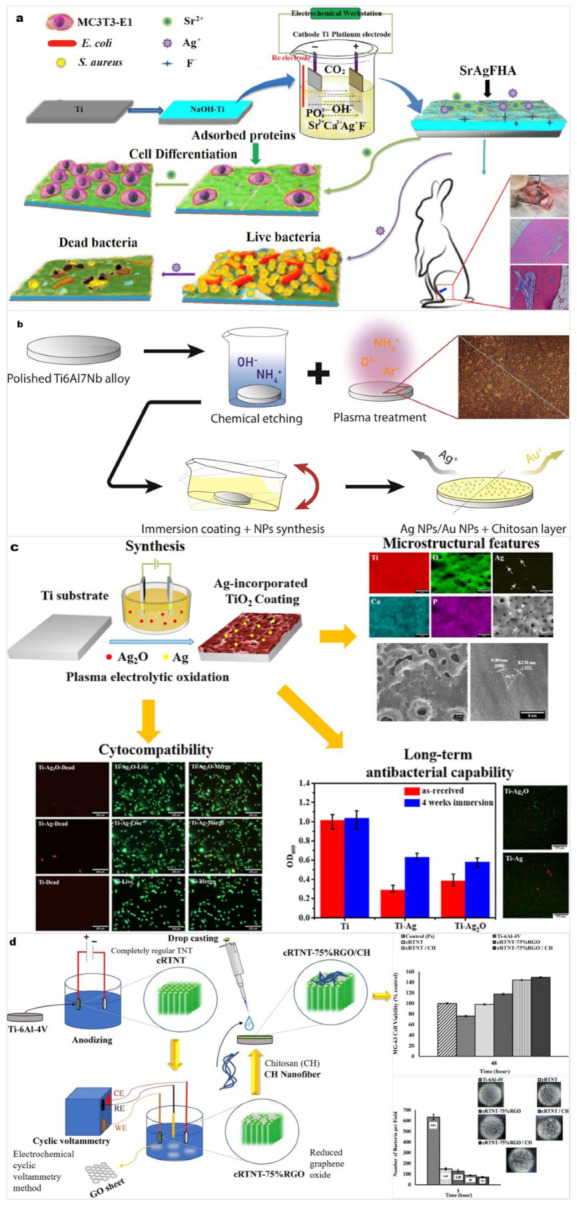
(**a**) Ag/Sr-codoped F-HA nanorod coatings on CP Ti plates using electrodeposition technique. (**b**) Chitosan layers with Au NPs/Ag NPs on chemical and plasma etched Ti-6Al-7Nb alloy. (**c**) Ag-doped TiO_2_ coatings on CP Ti using MAO in the electrolytes containing Ag or Ag_2_O nanoparticles. (**d**) Chitosan nanofibers (by electrochemical cyclic voltammetry method) and reduced graphene oxide (rGO) (by drop-casting) multifunctional surfaces on anodized Ti64, whose surface results in completely regular TNTs. Panel (**a**) reprinted with permission from [137] (Copyright 2020 Elsevier). Panel (**b**) reprinted under CC-BY-NC-ND license from [138] (2021). Panel (**c**) reprinted under CC-BY license from [139] (2021). Panel (**d**) reprinted with permission from [140] (Copyright 20 Elsevier).

Ti alloy implants are prone to potential complications, such as inflammation and bacterial adhesion after surgery. Bacteria that adhere to the surface of the implant accumulate in the hydrated polymer matrix to form a biofilm, which hinders the adhesion and growth of osteoblasts [135]. The impaired defense mechanism facilitates bacterial colonization and may lead to infection. To overcome this issue, covering the surface of an implant with a bacteriostatic coating is considered a feasible solution. To overcome bacterial resistance, it is necessary to use broad-spectrum antibiotics or inorganic antibacterial agents, such as metal ions and nanoparticles, on the surface of Ti alloy implants [136]. Figure 10 shows some new coatings that can slow the growth and spread of microorganisms of Ti and Ti alloy implants [137,138,139,140].

In the following section, we further summarize research advances in terms of functional antibacterial coating, factional, and antimicrobial surfaces on Ti alloy scaffolds. Electrochemical technologies such as electrodeposition, EPD, anodic oxidation, and MAO result in coatings containing different antibacterial components by the adjustment of the electrolyte level and a selection of voltage parameters. This is also linked to the joint effect of traditional methods such as alkali treatment, grit blasting, and the hydrothermal method. The research on antibacterial coatings by vacuum plasma spraying (VPS), dip-coating method, and chemical vapor deposition (CVD) are also provided as examples.

#### 3.3.1. Functional Antibacterial Coating

Studies have shown that the antibacterial effect of ion-doped HA largely depends on the concentration of the doping elements and the nature of the dopant itself [76]. Multi-ion co-doping produces better antibacterial and osseointegration effects than single-ion doping. Huang et al. [141] anodized Ti foils to prepare TNTs after alkali treatment, followed by the electrodeposition of nano-FAgHA. The dense and uniform FAgHA/TNT composite coating had a nanorod structure, and its corrosion resistance was improved by nearly two orders of magnitude. This coating exhibited high antimicrobial activity against *Staphylococcus aureus* (*S. aureus*), and its large specific surface area induced typical spherical apatite deposition. Thus, it showed good cell compatibility and was beneficial for cell osteogenesis in vitro.

Chitosan, a natural organic compound, can regulate the local concentration of drugs by controlling the thickness of the antibacterial coating that it forms [142,143]. Chitosan can be used to treat postoperative infection and inflammation, and to improve implant osseointegration, leading to rapid healing [7]. After alkali treatment of Ti, Zhang et al. [144] soaked the sample in heparin sodium and carboxymethyl chitosan solutions. Due to the large amount of −OH on the Ti surface after alkali treatment, the rough surface of Ti/OH could be tightly integrated with chitosan, and the sample exhibited excellent hydrophilicity and a contact angle of only 8°. The obtained coated Ti displayed no biological toxicity, and the inhibition rate of *S. aureus* and *Escherichia coli* (*E. coli*) reached 46–74%. Egemen et al. [145] used EPD to coat chitosan on Ti-6Al-4V after grit blasting. The morphology of the substrate after sandblasting affected the roughness of the chitosan coating and improved its wettability. It has been established that as the voltage increases, the deposition amount increases.

To improve the antibacterial and osteogenic properties of Ti, Huang et al. [146] prepared TNTs with a diameter of approximately 114 nm on Ti foils via electrochemical anodic oxidation, and used them to support the antibacterial drug norfloxacin, finally applying a methacrylate coating via free radical polymerization. Norfloxacin was released continuously for more than 168 h with only 34.4% burst release, and its antibacterial effect against *S. aureus* and *E. coli* persisted for up to five days. The antibacterial properties of MAO coatings can be significantly enhanced by the introduction of Ag, Cu, and Zn plasma. Zhang et al. [147] added CP Ti to a Ca(CH_3_COO)_2_·H_2_O solution for MAO, and then performed a hydrothermal treatment in Zn(CH_3_COO)_2_ solution. After heat treatment, the cytotoxicity was limited, and slow and constant Zn^2+^ release showed a 96.1% antibacterial rate against *S. aureus* with good biocompatibility. As Cu ions destroy bacterial cell membranes and interfere with DNA replication, they have received extensive attention [148]. Zhang et al. [149] explored the complementation/substitution of Cu and Zn ions in a doped titania coating with MATi plates in terms of cell activity/antibacterial properties. When doped alone, Zn and Cu ions did not significantly reduce cell viability. This indicated that although the concentrations of Zn^2+^ and Cu^2+^ were far lower than their half-maximal inhibitory concentrations, owing to their combined effects, cell proliferation still appeared to decrease, particularly at high Zn^2+^ concentrations. In terms of antibacterial properties, compared with Zn^2+^, Cu^2+^ was more effective in inhibiting the adhesion and reproduction of *S. aureus* at low concentrations. At a slightly higher concentration, the Zn^2+^ concentration was more critical for bacteriostasis. Van Hengel et al. [150] used MAO to explore the synergistic effect of Ag and Zn ions on the surface of selectively-laser-melted porous Ti implants. The minimum inhibitory concentration determined for the two ions was 1/120 that of the amount of individual silver ions required, confirming that the high antibacterial activity resulted from robust synergism; at the same time, the osteogenic behavior of pre-osteoblasts was enhanced.

Specific physical and chemical properties of the implant surface can also prevent bacterial colonization on the surface. For example, a crystallized anatase titania layer facilitates HA deposition [151], which significantly reduces bacterial adhesion and inhibits bacterial cell metabolism, providing satisfactory antibacterial performance and improved photocatalytic ability [152].

Other drugs (e.g., gentamicin, vancomycin, tobramycin, rifampicin, clindamycin, etc.) were also studied in different coatings. Table 2 lists some examples of the drug-containing surface modifications.

#### 3.3.2. Antimicrobial Surfaces on Ti Alloy Scaffolds

**Figure 11:**
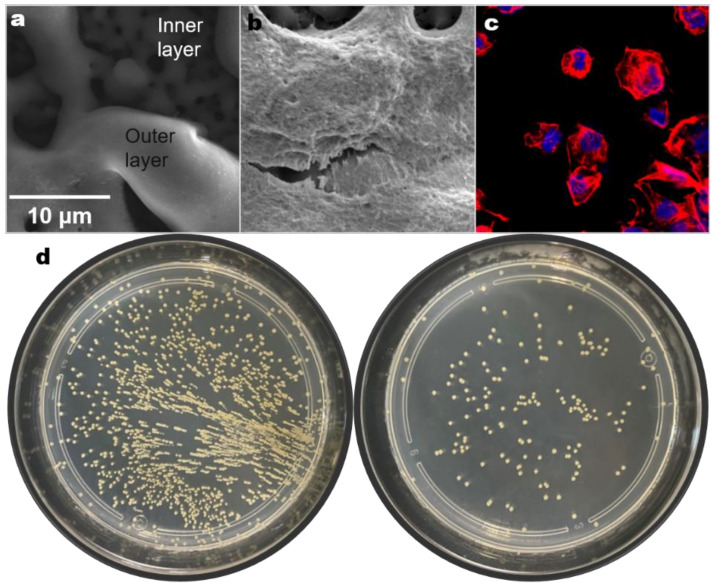
Antibacterial surface coating prepared by the authors (made by MAO). (**a**) Coating morphology. (**b**,**c**) Cell adsorption properties. (**d**) Antibacterial property against *S. aureus* on MAO-ed Ti used electrolyte without (**left**) and with (**right**) Ag.

Braem et al. [158] investigated the effect of surface roughness on bacterial proliferation on selectively-laser-melted dense and porous Ti and Ti-6Al-4V, employing polishing, machining, sand blasting, and VPS as surface modification methods. They found that porous and rough surfaces strongly influenced bacterial adherence, increasing the risk of infection at the implants, while chemical surface modification could effectively inhibit bacterial proliferation. To accelerate the vascularization and bone formation around selectively-laser-melted implants, the biochemical coating of porous implants can enhance their biological performance. Polycrystalline diamond (PCD) coatings have been developed in recent years, offering advantages such as mammalian cell growth promotion, apatite deposition strengthening, antibacterial capacity, appropriate chemical inertness, surface strength enhancement, and corrosion resistance. They are widely utilized in cardiovascular, orthopedic, and stomatology implants. Rifai et al. [159] utilized a dip-coating method to create nanodiamond (ND) coatings on selectively-laser-melted Ti. High-concentration (7.5% *w*/*v*) ND-coated Ti incubated a high density of human dermal fibroblasts and osteoblasts, while reducing *S. aureus* growth by 88%. Avoiding surface pretreatment, Rifai et al. [160] used CVD on selectively-laser-melted Ti64 to deposit a PCD coating with favorable adhesion and uniformity. The coating promoted the attachment and proliferation of normal Chinese hamster ovarian cells, improved osseointegration, increased bacterial adhesion resistance, and reduced *S. aureus* activity. The authors of the present study have also used MAO to prepare Ag-containing coatings on CP Ti prepared by SLM, and obtained an innovative, hierarchical porous surface containing both pores of 10 μm and 1 μm (Figure 11a). This surface has good cytocompatibility (Figure 11b,c) and significant antibacterial properties against *S. aureus* (Figure 11d).

## 4. Future Perspectives and Concluding Remarks

This review discussed various traditional and advanced surface modification methods that have been developed. The increasingly high surface performance requirements of Ti and Ti alloys for biomedical hard tissue replacement materials was also discussed. Appropriate surface modification methods should be selected to maximize the retention of the matrix properties of the various Ti materials being developed while meeting environmental requirements. Most of the technical results described and discussed in this paper are composite processes of more mature methods or the performance results of more advanced methods. Basic techniques, such as acid etching and sandblasting, are often used as the first step in the overall modification step in research, as they do not have the capability to perform significant modifications. Some advantages of advanced techniques compared to traditional techniques include stronger performance of the modified layer (advanced mechanical processing, PVD), improved modification efficiency (3D printing techniques using lasers such as LENS™, plasma spraying), and modification of the complex surfaces of porous lattices prepared using techniques such as SLM (electrodeposition, MAO).

3D printing technology, including LENS™, has great potential for application in surface modification. Due to the large laser power, spot size and powder size, it has high modification efficiency and robust capacity to build strong bonds between the modified layers and Ti and Ti alloy substrates. Depending on the research objectives, modified coatings with compatible thermal expansion coefficients, high hardness, strong wear and corrosion resistance, good electrochemical and tribological properties, strong fatigue properties and fracture toughness, and without adhesives and bio-toxicity can be obtained by changing the coating materials and printing parameters. Due to limited printing angles, LENS™ cannot compete with technologies such as electrodeposition when modifying the surface of complex implants. Its cost is also in the upper-middle range among the technologies. This technique also does not allow the preparation of coatings containing drugs to maximize biocompatibility.

Modified coatings constructed on the surface of Ti alloys through various techniques have led to significant improvements in the biocompatibility, osseointegration capacity, and antibacterial properties of the coated alloy, and a certain degree of progress has been achieved; however, owing to the demanding requirements of the complex in vivo physiological environment, in which implants manufactured using medical Ti alloys need to perform, many surface treatment methods have not been fully utilized.

In the future, work to improve surface modification technologies for Ti implants should be focused on the following research areas:

- The layered bone structure shown in Figure 1 is complex and this makes it difficult to achieve a similar layered design using surface modification techniques on the implant. Even if porous surfaces or biochemical agents are used, side effects such as coating failure and loosening of the implant cannot be completely avoided. In the future, customized bone-like alloys with hierarchical structure should be designed and prepared.
- As the real in vivo environment is complex, the corrosion and wear properties of the surface of Ti alloy implants need to be evaluated during long-term service;
- The majority of matrix materials are still based on CP Ti and Ti-6Al-4V, which have a high modulus of elasticity; therefore, further in-depth research is needed to determine the effects of alloying elements on the coating of new low-modulus β-Ti alloys;
- To reduce antibiotic overuse, desirable antimicrobial effects can be achieved by the incorporation of inorganic antimicrobial agents or chitosan. However, effective regulation of its dissolution rate and amount needs to be further investigated with regard to the balance between cytotoxic response and good antimicrobial activity.

Wear resistance, bioactivity, and antimicrobial properties are important for Ti and Ti alloys. Sometimes it can be difficult to improve both because approaches to improve wear resistance are often very different to approaches focused on improving biocompatibility, which may introduce biochemical macromolecules such as peptides and antibiotics, which hardly improve the wear resistance. Therefore, the focus should be first on improving the wear resistance before incorporating a bioactive layer that can be compounded and fixed. This can then meet the requirements of the complex environment of the implant. Future work on surface modification of medical Ti alloys should encompass multiple methods to maximize biofunctionality and make significant progress by improving the above-stated limitations, thus providing effective remedies to osteoporosis in an aging society.

## Figures and Tables

**Table 1:** Examples of surface modification of Ti and Ti alloys.

Alloys	Method	Modified Layer Thickness	Aims	Refs.
CP Ti	Atmospheric thermal oxidation + micro-arc oxidation (MAO)	∼2 μm (with a ∼70 μm oxygen diffusion layer)	The modified layer reduced the corrosion rate, and improved the tribocorrosion resistance, biocompatibility, and bonding between cells.	[48]
Ti64	Plasma spray coating	∼150 μm	The coating decreased the release of metal ions and improved corrosion resistance and microhardness values.	[49]
Ti-4Al-7Nb	High-speed milling	60–150 μm	The modified surface reduced the surface Young’s modulus and improved the hardness, corrosion resistance, and biocompatibility.	[50]
Ti-15Mo	MAO (in baths containing zinc)	3–15 μm	The coating increased surface the bioactivity and cytocompatibility and slightly improved the bacteriostatic effect.	[51]
Ti2448	Sandblasting, dual acid-etching, and alkali thermal treatment	∼3 μm	The surface roughness increased, and the surface wettability improved	[52]
Ti–29Nb–13Ta–4.6Zr (TNTZ)	Ultrasonic nanocrystal surface modification	∼150 μm (within the top ∼30 μm, a large amount of plastic deformation)	The nanostructured surface layer increased the wear resistance and cell adhesion area.	[53]

**Table 2:** Examples of drug-containing surface modification of Ti and Ti alloys.

Drugs	Methodology	Incorporating Structure	Results	Refs.
Gentamicin	Multilayers of gentamicin and polyacrylic acid obtained by LBL method on Ti64 treated by alkali-heating	Homogeneous cross-link multilayers’ deposition, gentamicin and polyacrylic acid coated layer-by-layer	Antibacterial rates compared to Ti64: 99.86% for *S. aureus* and 99.93% for *E. coli*	[153]
Vancomycin	Electrodeposition to coat HA/collagen/ vancomycin on Ti	Many small vancomycin crystals that aggregate and form larger islands	5 h antibacterial ratio (*S. Aureus*): ~97% 30 h drug release: ~85%	[154]
Tobramycin	Alkali-treated Ti screws with anodized surfaces soaked in Dulbecco’s PBS were soaked in tobramycin solution at 70 °C.	Tobramycin is deposited on a thin leaf-like HA coating that adheres tightly to the substrate.	Screws injected with *S. aureus* suspension were implanted into rabbits. The drug-laden group showed significantly lower signs of infection.	[155]
Rifampicin	Electrospinning process: the poly-caprolactone nanofibers loaded with HA nanoparticles and rifampicin were coated on acid-etched annealed Ti.	Regular and smooth fibers without beads or coagulations	Mechanical properties of nanocomposite scaffolds: increased threefold. Antibacterial: log reduction in the bacterial growth Drug release: 1 h: ~27%; 1 d: ~41%; 32 d: ~61%	[156]
Clindamycin	Hydrogel 3D bioprinting: hydrogels containing clindamycin were extruded on acid-etched Ti64	Rough surface by nanofibrillated cellulose filaments with furrows and without drug clusters	10-day degradation: ~57.8% (hydrogels without drug: 7-day: 100%) Drug release: 1 h: ~25%; 1 d: ~80%; 3 d: 100%	[157]

## Data Availability

Not applicable.
